# “It feels like an endless fight”: a qualitative study exploring healthcare utilization of persons with rheumatic conditions waiting for pain clinic admission

**DOI:** 10.1186/s12891-022-05808-6

**Published:** 2022-09-22

**Authors:** Nathan Blanchard, Simon Deslauriers, Jonathan Gervais-Hupé, Anne Hudon, Jean-Sébastien Roy, Sasha Bernatsky, Debbie E. Feldman, Anne Marie Pinard, Mary-Ann Fitzcharles, François Desmeules, Kadija Perreault

**Affiliations:** 1 Center for Interdisciplinary Research in Rehabilitation and Social Integration (Cirris), Centre intégré universitaire de santé et de services sociaux de la Capitale-Nationale, 525, boul. Wilfrid-Hamel, Quebec, G1M 2S8 QC Canada; 2 Faculty of Medicine, Université Laval, Ferdinand Vandry Pavillon, 1050 Av. de la Médecine, Québec, G1V 0A6 QC Canada; 3 École de Réadaptation, Faculté de Médecine, Université de Montréal, Montreal, Canada; 4 Centre for Interdisciplinary Research in Rehabilitation of Greater Montreal (CRIR), Centre intégré universitaire de santé et de services sociaux du Centre-Sud-de-L’Ile-de-Montréal, Chemin Hudson (Pavillon Lindsay), 6363, chemin Hudson (Pavillon Lindsay), Montréal, H3S 1M9 QC Canada; 5 Centre de recherche en éthique (CRÉ), 2910, Boul. Édouard-Montpetit 3ème étage, bureau 313, Montréal, H3T 1J7 QC Canada; 6 McGill University Health Centre (MUHC), 1650 Cedar Ave, Montreal, H3G 1A4 QC Canada; 7 McGill University, 845 Sherbrooke St W, Montréal, H3A 0G4 QC Canada; 8 Research Institute of the McGill University Health Centre (RI-MUHC), 1001 Decarie Blvd, Montréal, H4A 3J1 QC Canada; 9 Public Health Research Institute of Université de Montréal, 7101 avenue du Parc, Montréal, H3N 1X9 QC Canada; 10 Centre d’expertise en gestion de la douleur chronique du CHU de Québec-Université Laval, 2705, boulevard Laurier, #3412, Québec, G1V 4G2 QC Canada; 11 Maisonneuve-Rosemont Hospital (CRHMR) Research Center, 5415 Assomption boulevard, Montreal, H1T 2M4 QC Canada

**Keywords:** Health services utilization, Rheumatic diseases, Pain, Access, Wait time

## Abstract

**Background:**

Individuals living with a rheumatic pain condition can face delays in accessing pain clinics, which prevents them from receiving timely treatment. Little is known regarding their specific healthcare utilization in order to alleviate pain while waiting to obtain services in pain clinics. Hence, the aim of this study was to explore the perceptions and experiences of persons living with rheumatic conditions regarding healthcare utilization while waiting to access a pain clinic.

**Methods:**

In this qualitative descriptive study, semi-structured interviews were conducted with adults living with a painful rheumatic condition that reported either being waiting for admission in a pain clinic, having been referred but then denied pain clinic services, or having received services during the previous six months, in the province of Quebec, Canada. The interviews were transcribed verbatim, and an inductive thematic analysis was performed.

**Results:**

Twenty-six individuals were interviewed (22 women and 4 men; mean age 54 ± 10 years). Three themes were identified: 1) lacking guidance in identifying solutions to their complex and multidimensional needs, 2) struggling to obtain and maintain services due to systemic access barriers, and 3) displaying resilience through a search for accessible and sustainable self-management strategies.

**Conclusions:**

The current approaches and structures of health services fail to adequately answer the service needs of individuals experiencing painful rheumatic conditions. Important shifts are required in pain education, in increasing access to multidisciplinary approaches at the primary care level and in breaking down barriers individuals with chronic pain face to receive appropriate and timely care.

## Background

More than 54 million inhabitants in the U.S. [1], 4 million in Canada [2], 4 million in Australia [3] and 10 million in the U. K. [4] live with a rheumatic condition. In the U.S. and Canada, current projections estimate the prevalence of rheumatic conditions will nearly double within the next 20 years [1, 2]. Rheumatic conditions are not only recognized as a key population health issue because of their debilitating effects on function, but they are also a leading cause of pain [1–3]. The optimal management of persistent and severe pain and disability calls for a comprehensive, multifactorial, personalized approach, which is often offered in a pain clinic or a multidisciplinary pain treatment facility [5, 6].

However, delays in accessing services in a pain clinic can prevent patients from receiving timely treatment. Across Great Britain for example, in a study published in 2020, the average wait time from referral to treatment in a pain management clinic ranged from 6 to 112 weeks [7]. More than 87% of clinics reported that their average wait time exceeded 8 weeks [7] which is the International Association for the Study of Pain’s (IASP) benchmark for a non-urgent outpatient appointment for a persistent pain condition [8]. In Canada, Choinière et al. found that the median wait time in 2017–2018 was as high as 5.5 months, with some patients even having to wait up to 4 years before a first appointment in a public pain clinic [9]. In 2019, our team found that patients presenting a rheumatic condition had to wait a median time of just over 4 months before receiving services in a pain clinic in the Canadian province of Quebec [10]. While the Canadian health system offers publicly funded services to the population in a variety of settings that differ between provinces, ensuring access to these services in a timely and equitable manner has been a major issue for the population for years. Certain privately funded services are also available with high variability across Canada and for certain services only, including pain management services.

This waiting period has numerous consequences. Indeed, patients’ health-related quality of life and psychological status may worsen as a consequence of waiting for chronic pain treatment [11, 12]. In addition, the potential benefit of receiving treatment may be reduced or lost because of the wait. In a previous study from our team, patients diagnosed with a rheumatic condition who waited less than 2 months before their initial visit in a pain clinic reported superior improvements after 6 months of treatment, compared to those who had to wait more than 2 months. This was observed for the following variables: pain intensity, pain interference, and physical and mental components of quality of life [13]. Being faced with extensive delays before receiving treatments in a pain clinic has also been associated with high financial costs, as patients on wait lists may seek different therapeutic modalities and services to alleviate pain and prevent loss of function [14]. The types of healthcare resources used by patients on wait lists include medical treatments, physiotherapy, massage therapy and psychology [14]. However, specific healthcare utilization of persons living with rheumatic conditions who are waiting to obtain pain clinic services has not been documented.

Given that prolonged pain is associated with worsening health and high economic burden, an understanding of the perspectives of persons with rheumatic conditions waiting to receive treatment in a pain clinic may inform decisions to help better respond to their needs. Hence, the objective of this study was to explore the perceptions and experiences of persons living with rheumatic conditions regarding healthcare utilization while waiting to access a pain clinic.

## Methods

### Design

We employed a descriptive qualitative design [15–17]. This qualitative approach is increasingly used in the health field [17–19]. This study was part of a mixed-methods project exploring access to pain clinics for individuals living with a rheumatic condition [10, 13]. We conducted individual semi-structured interviews from June 2018 to February 2019. The Ethics Committee of the *Institut de réadaptation en déficience physique de Québec* (#EMP-2015–449) approved this study. The *COREQ* guidelines were considered for preparation of the current paper [20], while agreeing that the use of such guidelines can also be critiqued [19, 21].

### Study population

Study participants had to meet the following inclusion criteria: 1) be aged 18 and over, 2) have a diagnosis of a rheumatic condition (e.g., osteoarthritis, rheumatoid arthritis, fibromyalgia), 3) be able to communicate easily in French or English, and 4) having reported being either waiting for admission in a pain clinic in the province of Quebec, having been referred to a pain clinic but denied services, or having received services in a pain clinic during the previous six months. The purpose of the 6-month cutoff was to limit recall bias [22].

### Sampling and recruitment of participants

Different sampling strategies were employed to obtain a diversified group of individuals most likely to present varied perceptions and experiences regarding healthcare utilization [23–25]. First, a convenience sampling approach was employed. E-mails were sent to various organizations and patient associations (e.g., Arthritis Society, Quebec Association of Chronic Pain) and advertisements were posted in two pain clinics’ waiting rooms. Second, the snowball sampling method was used where recruited participants were asked to suggest other potential participants. Finally, a purposive sampling approach was used to target participants with sociodemographic characteristics that were underrepresented in our sample. Hence, we asked nurses and physicians from the various pain clinics to share the study invitation with potential participants who met specific criteria, which helped further include individuals responding to certain criteria (e.g., men or patients with osteoarthritis).

Persons who were interested in taking part in this study contacted the research team, either by telephone or by e-mail. Once potential participants' interest and eligibility were verified, an interview was scheduled. Written consent was obtained before the interview. The initial target was to recruit 30 patients with various profiles.

### Data collection

A semi-structured interview guide was developed based on previous studies on access and the results of the first part of our mixed-method project, which was a database study using the Quebec Pain Registry [10, 13]. Furthermore, the interview guide was continually adapted throughout data collection via an iterative process [26]. The questions focused on the participants’ perceptions and experiences regarding access to a pain clinic, their healthcare utilization, the impact of the waiting time on their condition, possible avenues for improvement (see previous paper [27]), as well as healthcare utilization, which is the focus of this paper. The interviews, which lasted on average 45 min, were recorded using a digital audio recorder and were transcribed verbatim. Notes were taken during and after each interview as an initial step contributing to the analysis process. The interviews took place over the phone or in-person at the research center or the participant’s home, based on participants’ preference.

### Patient and public involvement

Two persons living with a painful rheumatic condition were involved in the design stage of the study, more specifically within the preparation of the interview guide and planning of the interview process. The two persons were chosen as representing potential study participants, as they would have met inclusion criteria. However, they were clearly informed at initial contact that their contributions were framed within study preparation, not data collection. The interview process conducted with them was viewed as a step aiming to improve the richness of the data that would be collected with recruited participants. Hence, the two persons took part in a pilot interview and were then asked to provide personal feedback on the content covered within the interview guide (e.g. in relation with the study objectives) and on the interview process. Undertaking these two interviews allowed the study team to refine the questions and data collection procedures.

### Data analysis

Data were analyzed using thematic analysis [28–30]. Thematic analysis has been approached and operationalized within diverse ontological, epistemological and theoretical viewpoints and there is a clear need to make these underpinnings explicit [30, 31]. The following positions and assumptions were intended to guide our analyses: 1) analysis was viewed as a subjective and interpretative process, 2) the team members involved in the analysis contributed their own skills, viewpoints and experiences to the work, 3) analysis was considered an inductive and iterative process that reflected repeated engagement with the data by those involved [31]. Furthermore, our approach to qualitative inquiry was intended to be conducted within a relativist/constructivist ontological stance, one of the stances described by Terry et al. [31]. Nonetheless, we acknowledge that our team brought together multiple viewpoints and experiences in conducting qualitative thematic analysis and that the overall process was also most likely influenced by a realist stance [31].

Data analysis was initiated simultaneously to data collection. The initial analysis allowed to orient the recruitment process in order to ensure maximal variation in perspectives and to adapt the interview guide [32]. As described by Miles et al., the first cycle of the coding process focused on coding words, sentences and paragraphs derived from the interviews [28]. The data were read over several times to promote deep engagement [33]. In line with a more reflexive and inductive approach to thematic analysis, no a priori codes or conceptual framework were used [21, 31]. Codes were then sorted into different categories in a structured but flexible arrangement [28]. As the data collection and analysis processes advanced, the code list eventually grew into a thematic coding tree: codes were added, displaced to other categories, combined, or subdivided [28]. The goal of the second cycle of coding was to obtain a higher level of analysis through identifying relationships and explanations of the most relevant themes [28]. Guided by our intention to ensure rigor and credibility of the data analysis and suggested by some [34], NB and JGH independently coded three interviews in order to share their perspectives regarding a common set of data and the coding and theme development stages of the analysis process. This did not lead to verification and measuring of coding reliability, but was rather followed by numerous discussions between NB, JGH, AH and KP before agreeing on a final thematic coding tree. NVivo 12 (QSR International Pty Ltd.) was used to facilitate the thematic analysis [28]. The most relevant citations were translated from French to English by a bilingual individual (and translations were verified by a second team member). They are included in the results to exemplify the identified themes.

## Results

### Study sample

Twenty-six individuals took part in an interview, 22 women and four men. Sixteen interviews were conducted over the phone while 10 others were done in-person. The mean age of the participants was 54 years (standard deviation = 10 years), with a range of 39 to 82 years (see Table 1). Close to half (46.2%) of participants had a university degree. Only 11.5% of participants were actively employed and 42.3% were on sick leave, either temporarily or permanently. The household income for 50% of participants was below 40,000 Canadian dollars (CAD), knowing the average annual income in Quebec amounted to 45 000$ in 2018 [35]. Sixty-nine percent of participants had been waiting for more than 12 months to access services in a pain clinic.

**Table 1:** Participants' sociodemographic characteristics

Characteristics	n (%)
Gender	
Women	22 (84.6)
Men	4 (15.4)
Age (years)	
< 40	3 (11.5)
40–55	12 (46.2)
56–70	10 (38.5)
> 70	1 (3.8)
Education (highest degree obtained)	
Elementary	1 (3.8)
High school	8 (30.8)
College	5 (19.2)
University	12 (46.2)
Employment status	
Employed (full-time or part-time)	3 (11.5)
Sick leave / on temporary disability	6 (23.1)
Sick leave / on permanent disability	5 (19.2)
Retired	10 (38.5)
Other	2 (7.7)
Household income (CAD^a^)	
≤20,000	5 (19.2)
20,001–30,000	5 (19.2)
30,001–40,000	3 (11.5)
40,001–50,000	2 (7.7)
50,001–70,000	3 (11.5)
> 70,000	7 (26.9)
Missing	1 (3.8)
Diagnosis^b^	
Fibromyalgia (FM)	18 (69.2)
Osteoarthritis (OA)	11 (42.3)
Ankylosing spondylitis	6 (23.1)
Rheumatoid arthritis (RA)	3 (11.5)
Spondylodiscitis	1 (3.8)
Pain clinic wait list status	
On a wait list	16 (61.5)
Has consulted	6 (23.1)
Removed from a wait list	2 (7.7)
Consulted before and back on wait list	2 (7.7)
Wait time duration^c^	
< 6 months	5 (19.2)
6–12 months	2 (7.7)
1–2 years	9 (34.6)
2–5 years	8 (30.8)
> 5 years	1 (3.8)

^a^ CAD: Canadian dollars

^b^ More than one answer was possible

^c^ Including patients on a wait list, those who consulted and those who were excluded

### Perceptions and experiences regarding healthcare utilization

**Fig. 1:**
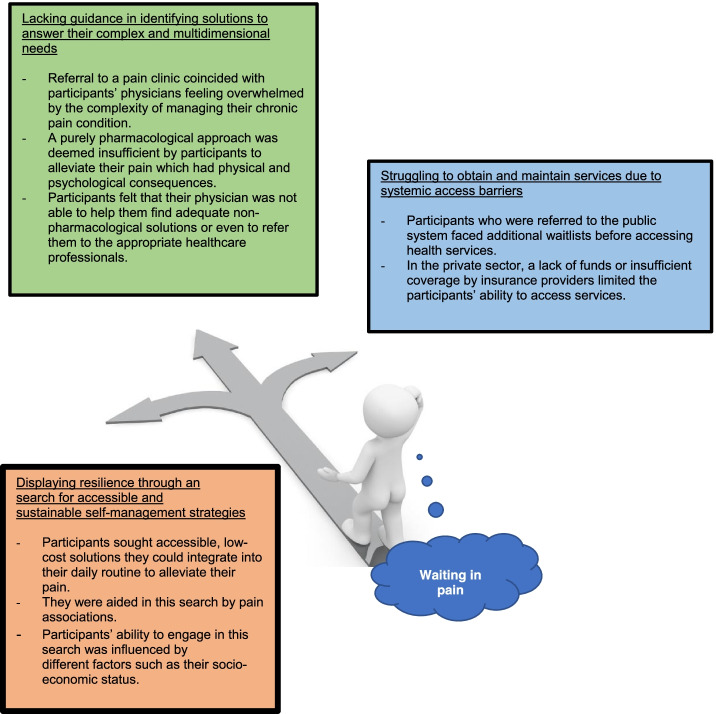
Main themes identified regarding healthcare utilization while waiting for admission in a pain clinic

Participants’ perceptions and experiences regarding healthcare utilization while waiting to obtain services in a pain clinic fell under three themes: 1) lacking guidance in identifying solutions to answer their complex and multidimensional needs, 2) struggling to obtain and maintain services due to systemic access barriers, and 3) displaying resilience through a search for accessible and sustainable self-management strategies. The three themes reflect common experiences participants had regarding their healthcare utilization and are interrelated. Figure 1 presents key ideas falling under each theme.

#### Lacking guidance in identifying solutions to answer their complex and multidimensional needs

The first theme that was identified describes the participants’ perceptions of not being properly guided in identifying pain management interventions and in answering their needs. In that regard, participants all referred to their primary care management by a family physician. A majority of participants felt that, aside from adjusting their medications, their primary care physicians were not able to help them find the proper resources and strategies to ease their pain.

To start, primary care physicians mainly addressed their patients’ pain condition by prescribing medications. However, many participants mentioned how, over time, the number of different medications they had to take, the need for constant adjustments of dosages, the physical and psychological side effects as well as the phenomenon of tolerance often contributed to negative experiences. One participant explained:

“For sure, medication, I would like not to take any of it, I hate it, but I don’t have a choice because when we try to remove it, I pay the price’’ (Participant 4, woman, in her 60s, fibromyalgia, waiting 3-5 years).

Another participant stated: “We are on drugs all the time. It’s a lease for life” (Participant 7, woman, in her 30 s, multiple rheumatic conditions, waiting 1–2 years). The participants also expressed how their pain medication alone was not sufficient to manage their chronic pain condition. After trying several combinations of medications, one participant began to ask herself:

“Am I still taking them for nothing? Is there something for my health in taking them? Am I solving one problem only to cause other problems?” (Participant 6, woman, in her 50s, multiple rheumatic conditions, waiting < 1 year).

In another situation, a participant was prescribed antidepressants over and over again, but she explained that they were not the right solution for her:

“I am taking some right now, but it doesn’t change anything. […] It does not stop me from crying, it doesn’t stop me. It’s not something I need.’’ (Participant 7, woman, in her 30s, multiple rheumatic conditions, waiting 1-2 years).

Most of the time, pain was present over several months or even years before participants were referred to a pain clinic. In many cases, the moment of referral coincided with their primary care physicians feeling overwhelmed by the complexity of their patients’ conditions or even with the participants judging that their physicians could not help them adequately anymore. One participant stated:

“[My family doctor] even told me he could not do anything for me the last time I saw him. In my opinion, it is unacceptable to tell that to a patient’’ (Participant 3, man, in his 60s, osteoarthritis, waiting 3-5 years).

This breaking point was the result of the limited options available to primary care physicians with regards to treating chronic pain. Even though the referral to a specialized pain clinic was perceived as a step in the right direction, the participants still needed someone to guide them during the lengthy waiting period before their pain clinic admission. The limitations of the pharmacological approach led the participants to seek solutions which went beyond the objective of alleviating their pain, since the multidimensional nature of their chronic pain condition also required treatment for their physical and psychological well-being. One participant expressed the specific need felt by many participants to be guided during the waiting period:

“You know, I would like to have someone coach me. […]. Do I need a psychologist or a psychiatrist? Do you need a chiropractor or a physio? I don’t know anymore. I try everything. I’m the one who’s going by trial and error” (Participant 13, woman, in her 50s, multiple rheumatic conditions, waiting < 1 year).

Another participant also stated how she felt her primary care physician was limited in his capacity to guide her:

“The doctor can do two things for you: give you medication then give you papers to go see other specialists and […] a paper for physiotherapy, a paper for the chiropractor, a paper for ... There are a lot of papers he can give you […]. He can’t do anything else; he is a family doctor. He won’t give you a massage and then he won’t give you exercises; it’s not his job. Besides, I asked him [for exercises] and he said he had no idea” (Participant 4, woman, age 67, fibromyalgia, waiting 3-5 years).

Hence, while participants expected diverse options in order to meet their complex and multidimensional needs, they felt their primary care physicians, on whom they relied for this, lacked knowledge in guiding them to obtain such interventions. Still, some participants obtained a referral to healthcare services in the public or the private sector, including psychology, psychiatry, kinesiology, occupational therapy, and physiotherapy, as well as consultations with medical specialists such as rheumatologists. Systemic access barriers in the private and public healthcare sectors nonetheless led to several different challenges for our participants in their search for solutions to alleviate the consequences of their chronic pain condition.

#### Struggling to obtain and maintain services due to systemic access barriers

A second theme was the difficulties faced by the participants, during their waiting period, to obtain and maintain healthcare services in both the public and the private healthcare sectors.

One of the main challenges faced by participants was being placed on waiting lists to obtain publicly funded healthcare services, in addition to already being on another wait list for admission in a public pain clinic. When participants received a referral to consult a healthcare professional in the public sector, they faced lengthy delays in accessing these services. One participant waiting to see a psychologist mentioned: “I don’t know how many years long the wait list is. There is always a wait list” (Participant 9, man, in his 50 s, fibromyalgia, waiting 1–2 years). Another participant described the additional wait times by saying:

“My perception is that, I would say, I am tired of being in this system. Do you understand? To wait. […] It feels like an endless fight” (Participant 13, woman, in her 50s, multiple rheumatic conditions, waiting < 1 year).

One participant explained:

“The healthcare system has almost become inaccessible. You can pay money to get services in the private sector. If you go to the private sector, for sure it’s faster, but if not, you are on waiting lists and it takes an enormous amount of time or you’re downright unable to see anyone” (Participant 21, woman, in her 60s, fibromyalgia, waiting 3-5 years).

Therefore, the participants often had to turn to the private sector to obtain services, in what they considered a more acceptable timeframe. The private sector was also an entry point to obtain services that were not offered within the public system such as those offered by naturopaths, massage therapists, chiropractors, and acupuncturists.

However, certain individuals had more difficulty in obtaining or maintaining healthcare services in the private sector during their waiting period: those with limited insurance coverage, those having a low socio-economic status and those having a diagnosis of fibromyalgia. Consulting healthcare professionals in the private sector required having insurance plans to help cover fees for these services, although most coverage was insufficient to maintain services. Thus, participants had to choose which services to keep or discontinue:

“Of course, because you know, when you are retired, you have a budget and when it costs you 110$ for a psychologist, 90$ for a kinesiologist, 60$ for physiotherapy and for chiro and 50$ for the massage therapist, you have to choose” (Participant 4, woman, age 67, fibromyalgia, waiting 3-5 years).

Another participant stated:

“You see, my insurances renew at the beginning of January and right now I have already used up all my treatments. So, either I stop, or I pay out of my own pocket” (Participant 22, woman, in her 50s, multiple rheumatic conditions, waiting 1-2 years).

Over time, some participants who had partial coverage by insurance plans were not able to pay their share because of a loss of income due to their pain condition. Waiting in pain extended their work disability status which limited their earnings, and hence, their ability to obtain services in the private sector.

Some participants did not have any insurance plans or could not afford to pay for these services on their own. This was especially true for participants with a lower socio-economic status. One participant explained: “I don’t have the money. I make 25,000$ per year” (Participant 3, man, in his 60 s, osteoarthritis, waiting 3–5 years). Another participant, who was on social security mentioned:

“Anyway, even if I found the money, which would be very unlikely, then social security would tell me ‘How did you pay for this?’. What I am given is last resort help, I am supposed to pay my rent, my bills, my food, and that’s it” (Participant 23, woman, in her 40s, fibromyalgia, waiting 1-2 years).

Obtaining healthcare services in the private sector was also impossible for some participants who were denied any disability coverage by their insurance providers, especially participants with fibromyalgia. One participant stated:

“There is a big gap, a judicial gap because […] it is hard to make fibromyalgia recognized by the [Quebec Pension Plan]. […]. The insurance providers do not want to support us, do not want to cover us. […] Let’s be clear, they do not want to pay after one year. So, they do everything to make us go back to work” (Participant 7, woman, in her 30s, multiple rheumatic conditions, waiting 1-2 years).

In summary, the additional waitlists in the public healthcare sector combined with the difficulties related to obtaining sufficient coverage in the private sector left several individuals struggling to obtain healthcare services during their waiting period.

#### Displaying resilience through a search for accessible and sustainable self-management strategies

The third theme addresses the participants’ search for accessible and sustainable self-management strategies**.** Despite previously discussed obstacles, participants still sought to find accessible and affordable strategies for pain self-management that they could use regularly to meet their needs.

Many participants found the help they sought through organizations like the Quebec Chronic Pain Association and the different branches of the Fibromyalgia Association of Quebec. One participant explained:

“All I got is the information I received from the Chronic Pain Association: lectures, discussion workshops. I participated in a lot of these activities, and I thank them for their support, and it is them who have done the most work to help me. It’s not the healthcare system” (Participant 3, man, in his 60s, osteoarthritis, waiting 3-5 years).

In one case, one of these organizations served as a path to obtain services by offering their members access to a massage therapist for a reduced-price. These associations allowed people suffering from chronic pain conditions to engage with their peers, learn new strategies and feel less isolated regarding their pain management: One participant mentioned:

“With the association of fibromyalgia, we listen to many conferences, and also talk a lot about pain, treatments, what works, what doesn’t” (Participant 28, woman, in her 60s, multiple rheumatic conditions, waiting < 1 year).

However, many participants were not informed about these organizations by healthcare professionals and came across their existence either on their own or by pure coincidence. This is why one participant suggested:

‘‘When people meet their family doctor, and have been diagnosed […], their family doctor should refer them to associations in order to find help. [Without these associations], there are many people who have nothing’’ (Participant 25, woman, in her 50s, fibromyalgia, waiting 1-2 years).

Either on their own or through these associations, participants learned about the benefits of different alternative and complementary therapies. Many individuals found pain relief through martial arts (ex. Tai-chi, qigong), yoga, relaxation techniques (visualization, distraction and breathing exercises), the use of heat, stretching, and aquafit. A number of participants among our sample talked about the importance of integrating these self-management strategies into their daily routines, otherwise they would feel the impacts of neglecting them:

“If I don’t do my qigong for a week, I notice, my neck stiffens, my spine, my elbow. Exercises, if you don’t do them, it will get worse” (Participant 30, man, in his 40s, ankylosis spondylitis, waiting 3-5 years).

Leading this search for self-management strategies is a display of these participants’ resilience. One participant described this process as follows:

“Trying to find and asking for help. That’s the problem. We ask for some and we don’t get a lot in return. But you must never stop asking for help” (Participant 4, woman, age 67, fibromyalgia, waiting 3-5 years).

Another participant who had been waiting less than a year for admission in a pain clinic explained:

“I went through a lot of pain and then I felt like I was lost in it. Now I have the character to say, I’m going to stand up for myself, I’m going to find solutions on my own. This is where I am at. But getting there, I had some pretty tough times” (Participant 6, woman, in her 50s, multiple rheumatic conditions, waiting < 1 year).

However, all participants did not have the same opportunity to engage in the process of finding solutions. Different factors like their financial capacity influenced the participants’ search for help. As explored in the second theme, those with more financial means could obtain services promptly. They could also consult a broader range of healthcare professionals only accessible in the private sector and receive a greater number of treatments. Many participants with a higher socio-economic status even expressed that they had tried everything. One participant had travelled to different cities. Nevertheless, participants with a low socio-economic status were not able to engage in such a process in order to find the right services to alleviate their pain. Hence, these participants tended to place their hope in the pain clinic and waited for their admission eagerly. One participant who was on social security and could not afford services in the private sector stated:

“I live in low-rent housing because I have not worked enough in Quebec to have access to a disability pension. So I am on social security. I take what [services] I am offered” (Participant 9, man, in his 50s, fibromyalgia, waiting 1-2 years).

The same participant went on to say:

“What can [the pain clinic] do [during the waiting period]? I understand that they are booked. [… ] So I get that even though I’m in pain, I’m just going to have endure the waiting patiently” [… ]. But sometimes I feel like a number”(Participant 9, man, in his 50s, fibromyalgia, waiting 1-2 years).

Conversely, the pain clinic was viewed less as a necessity for many who had access to more options for treatment. As one participant with more financial means explained:

“Look, at first, I had really high expectations [towards the pain clinic]. Now I have less and less because, like I’m telling you, I can't, we can't wait. You can't be waiting for something all the time. I stopped waiting. […] Now, I take care of myself. Now, I have cleared my head and started from scratch (Participant 16, woman, in her 40s, fibromyalgia, waiting 1-2 years).

Another participant was considered as ‘‘Wonder Woman’’ by her friends and relatives for her ability to handle simultaneous challenges and take care of herself. Her perseverant attitude in finding solutions was reflected through her own words:

“[You] need to find then ask for help. That’s the problem. We ask for help but we don’t get much in return. But you must never stop asking. Sometimes, [even though] it’s bothersome, you need to harass people. […] And I admit that asking is not easy and even sometimes, it is humiliating" (Participant 4, woman, age 67, fibromyalgia, waiting 3-5 years).

For some participants who had the physical, mental, social, and financial resources, this reflected a shift towards self-management and empowerment.

Another instance where participants had to search for strategies on their own was when they reported being denied access to or removed from a pain clinic’s waiting list. This happened to some participants suffering from fibromyalgia. One participant with fibromyalgia summed up the challenges faced by individuals with this diagnosis in the current system:

“There is no help for fibromyalgia. No help at all. When I tell you none, we are stuck between two chairs. We don’t have access to the pain clinic, […] Because when you are diagnosed with fibromyalgia and then you can't work, and you don't qualify for any government program, it gets tough.’’ (Participant 27, woman, in her 40s, fibromyalgia, was denied being placed on waiting list).

## Discussion

This study explored the perceptions and experiences of persons living with rheumatic conditions regarding healthcare utilization while waiting to access a pain clinic.

The first identified theme was the participants’ lack of guidance in identifying interventions to answer their complex and multidimensional needs. Participants relied on their primary care physicians but felt that these were lacking in ability to manage pain, notably in identifying non-pharmacological approaches and referral to appropriate healthcare professionals. One reason may be that primary care physicians may not be well-trained to address chronic pain. In a study investigating the designated hours for pain teaching in Canadian healthcare faculties found that a mean of 16 h was integrated into the entire medical curricula, compared to 41 h received by physiotherapy students and 87 h for veterinary students [36]. The lack of pain management training in medical schools has also been reported in the US [37] and UK, where pain education has been described as “woefully inadequate given the burden of pain in the general population” [38] (p. 794). Recent work indicates that medical education seems to focus more on preventing the misuse of opioid narcotics rather than giving adequate attention to non-pharmacological approaches, nonopioid medications, interdisciplinary training, and physicians’ ability to provide empathetic care [39]. These discrepancies contribute to fueling the perception that “chronic pain patients are difficult, drug seeking and manipulative” [40] (p. 778). In a 2018 study, medical students at different stages of training expressed a negative view of handling these patients [40]. Inability to cure pain conditions, as well as being told by mentors that there was limited educational value in seeing persons living with chronic pain conditions, were among the reasons that contributed to their negative perceptions [40]. Primary care physicians describe being challenged in managing chronic pain due to struggles their patients face with poverty, mental health problems and addiction [41], observations that also resonate with our findings. Carr and Bradshaw recommended changing medical pain curriculum to further include consideration of interpersonal and social aspects of chronic [42]. As another barrier to appropriate guidance felt by our participants, healthcare providers don’t always adhere to guidelines in order to select recommended non-pharmacological and pharmacological pain treatment [43, 44]. Lack of knowledge about potential benefits of non-pharmacological interventions could also explain low referral for such services [44].

These results also reflect how participants regard their primary care physician as their main gatekeeper in managing their chronic pain. However, their expectations place a heavy burden on primary care physicians who also face challenges related to being overburdened with patient volume; and treating chronic pain is not only difficult and complex but potentially can seem frustrating and unrewarding [40]. In addition, primary care physicians are met with barriers when referring patients to publicly funded services, as these are often limited and inaccessible [44]. Certain initiatives have already been undertaken to improve interprofessional collaboration in primary care settings [44, 45] particularly with physiotherapists who may be well suited to lead pain management in new primary care [46].

The second theme specifically highlights the systemic barriers that participants faced in accessing services, both in the public healthcare and private sectors. In the public sector, participants faced wait times that added to those already experienced before accessing pain clinics. Underfunding of multidisciplinary treatments in primary care may explain why persons experiencing chronic pain face prolonged waiting times [47]. Facing delays is a well-known obstacle for persons with chronic pain who have to navigate “in a healthcare system that is better equipped to fix broken bodies than address suffering without obvious pathology” [48] (p.204). Indeed, acuity of conditions has been identified as a leading factor for wait list prioritization [49].

In the private sector, a lack of funds or insufficient insurance coverage explained why participants were not able to obtain or maintain health services. It has been well documented that chronic pain is more prevalent in individuals with lower socio-economic status [50]. Around 40% of our participants had an annual household income inferior to 30,000 CAD which is just above the viable income threshold [51]. Participants with a higher socio-economic status are more able to afford these services through private insurance plans or by paying out-of-pocket [41].

The third theme highlights resilience in participants’ search for self-management pain strategies. Some participants were supported in this search by patient associations, through education and support groups. There seems to be uncertainty among healthcare professionals and the public, about the role of patient associations in this regard. Indeed, there is also a paucity of literature exploring the roles of such associations and how they integrate within the healthcare environment in order to better support chronic pain patients. In addition, our study showed that individuals with a higher socio-economic status tended to view the pain clinic less as a necessity than those with less financial means. The former could afford to seek and obtain a wider array of solutions for pain relief. Participants with a lower socioeconomic status did not appear less resilient or proactive in their search for pain strategies than participants with a higher socioeconomic status. They just did not have the resources to take part in a long process of trial and error in trying different pain-relieving solutions, as others have highlighted [52]. While being an extraverted person [53], seeking positive social interactions [54], and securing social support may all lead to more resilience in the presence of chronic pain, an individual’s socioeconomic status may hamper the ability to take part in a lot of endeavors to manage their pain [52, 55]. This observation also highlights the importance of taking into account social determinants of health in chronic pain management. Being resilient is often viewed as a personal trait where one is able to adapt as well as remain flexible and optimistic in order to weather their pain. However, as presented in our results, individual and environmental factors can limit one’s opportunity to adapt.

Our sample included mainly relatively highly educated individuals. Higher education has been associated with superior healthcare literacy [56] which may increase an individual’s engagement in their pain management. In an Austrian study led by Köppen et al., individuals presenting chronic pain who had higher healthcare literacy showed less pain intensity, possibly suggesting better pain management and coping strategies [56]. Those with low healthcare literacy tend to delay or even forego needed care, and have more difficulty in finding healthcare providers [57]. Ironically, it is patients with fewest resources who appear to have worst access to optimal pain management.

The Canadian Pain Task Force recently reported several subgroups of the population who are “disproportionately impacted by pain” [58] (p. 16), because they face more barriers to care and higher occurrence and gravity of their chronic pain conditions. These may include for instance women, seniors, certain ethnic and racialized communities, indigenous peoples, as well as people who are sexually and gender diverse. This Task force advocates for novel strategies to address these disparities such as the “expansion of specialized services for painful conditions predominantly by women” [58] (p. 18), such as fibromyalgia which does not fit into the biomedical model of health care and is often treated with doubt as if the disease did not exist [58, 59]. As explored in our results, participants with fibromyalgia, all women, faced difficulty in accessing health services in the private health sector and some were removed from publicly funded pain clinic wait lists.

### Limitations

Our study has limitations. First, although part of the interview guide focused on healthcare utilization during the waiting period before admission in a pain clinic, the interviews did not exclusively focus on this subject. This may have prevented a deeper understanding of certain aspects related to healthcare utilization. Also, this study allowed to explore the perceptions and experiences of persons waiting for services, a majority having fibromyalgia, which may not reflect the lived experiences in other pain-related conditions. Our sample also lacked diversity regarding certain characteristics such as gender and level of education. A further limitation in the current study is that data on ethnicity was not available. This should have also been systematically collected. Indeed, all these factors have been found to impact the experience of pain and healthcare access [56, 58–60]. Another limitation is while participants had different backgrounds regarding service access (i.e. were either waiting for treatment, denied treatment, or received treatment), and that certain differences were highlighted between subgroups of participants, the data were not systematically analyzed through this particular lens. In addition, while a patient partner was involved in data collection preparation, none was involved in the overall conception of the study, nor in other steps, such as data analysis and interpretation. This would have been very helpful and could have strengthened the overall research process. The overall quality of our qualitative approach was also most likely influenced by the fact that our work was situated within a larger mixed methods study. Indeed, the research team faced tensions between carrying out, on the one hand, a more technical approach to thematic analysis (inspired by realist and post-positivist orientations, most likely influenced by the quantitative part of the overall project) and a more reflexive approach to thematic analysis which we aimed for [30, 31]. Furthermore, most participants had or had had a primary care physician during their waiting period. Hence, our study may not well reflect the experiences of persons who also lack access to a primary care physician. A more complete understanding of all the challenges surrounding pain clinic admission for persons living with rheumatic conditions would have also required conducting interviews with other relevant actors such as primary care physicians, physician specialists, and pain clinic staff and administrators. Finally, this study was conducted before the COVID-19 pandemic and the experiences of persons living with rheumatic conditions and chronic pain may have changed since.

## Conclusions

In conclusion, this study explored the perceptions and experiences of healthcare utilization while waiting to access a pain clinic for individuals living with rheumatic conditions. Based on our results, these individuals lack guidance from their primary care physicians in identifying multimodal solutions. They also face systemic access barriers to services both in the public and in the private sector. However, participants in this study also showed resilience in leading a search for self-management strategies to ease their pain.

Chronic pain has been compared to a ‘‘silent epidemic’’ [61]. The consequences of having millions of individuals living in pain waiting for services inevitably impacts societies. Through their voices, the participants in this study have exposed shortcomings of the current system with regards to addressing chronic pain and answering persons’ needs. Important shifts may be needed in pain education, in increasing multidisciplinary treatment, notably at the primary care level; and in breaking down barriers faced by persons experiencing chronic pain in accessing appropriate and timely care, as well as in helping them find the appropriate internal and external resources necessary for self-management.

## Data Availability

The datasets generated and analysed during the current study are not publicly available but are available (de-identified) from the corresponding author on reasonable request.

## Abbreviations

CAD: Canadian dollars
